# A Green, Simple and Facile Way to Synthesize Silver Nanoparticles Using Soluble Starch. pH Studies and Antimicrobial Applications

**DOI:** 10.3390/ma14164765

**Published:** 2021-08-23

**Authors:** Bogdan Pascu, Adina Negrea, Mihaela Ciopec, Narcis Duteanu, Petru Negrea, Nicoleta Sorina Nemeş, Corina Seiman, Eleonora Marian, Otilia Micle

**Affiliations:** 1 Faculty of Industrial Chemistry and Environmental Engineering, Politehnica University of Timisoara, 300006 Timisoara, Romania; ioan.pascu@upt.ro (B.P.); petru.negrea@upt.ro (P.N.); 2 Renewable Energy Research Institute, Politehnica University of Timisoara, 300501 Timişoara, Romania; 3 Faculty of Chemistry, Biology, Geography, West University Timisoara, 300115 Timisoara, Romania; corina.duda@e-uvt.ro; 4 Faculty of Medicine and Pharmacy, University of Oradea, 410068 Oradea, Romania; marian_eleonora@yahoo.com (E.M.); micleotilia@yahoo.com (O.M.)

**Keywords:** colloidal silver nanoparticles, starch, ultrasonic disruption, pH, antimicrobial tests

## Abstract

Along with the progress of nanoscience and nanotechnology came the means to synthesize nanometric scale materials. While changing their physical and chemical properties, they implicitly changed their application area. The aim of this paper was the synthesis of colloidal silver nanoparticles (Ag-NPs by ultrasonic disruption), using soluble starch as a reducing agent and further as a stabilizing agent for produced Ag-NPs. In this context, an important parameter for Ag-NPs preparation is the pH, which can determine the particle size and stability. The physical-chemical behavior of the synthesized Ag-NPs (shape, size, dispersion, electric charge) is strongly influenced by the pH value (experiment being conducted for pH values in the range between 8 and 13). The presence of a peak located at 412 nm into the UV-VIS spectra demonstrates the presence of silver nano-spheres into the produced material. In UV/VIS spectra, we observed a specific peak for yellow silver nano-spheres located at 412 nm. Samples characterization was performed by scanning electron microscopy, SEM, energy-dispersive X-ray spectroscopy, EDX, Fourier-transform infrared spectroscopy, and FT-IR. For all Ag-NP samples, we determined the zeta and observed that the Ag-NP particles obtained at higher pH and have better stability. Due to the intrinsic therapeutic properties and broad antimicrobial spectrum, silver nanoparticles have opened new horizons and new approaches for the control of different types of infections and wound healing abilities. In this context, the present study also aims to confirm the antimicrobial effect of prepared Ag-NPs against several bacterial strains (indicator and clinically isolated strains). In this way, it was confirmed that the antimicrobial activity of synthesized Ag-NPs was good against *Staphylococcus aureus* (ATCC 25923 and *S. aureus* MSSA) and *Escherichia coli* (ATTC 25922 and clinically isolated strain). Based on this observation, we conclude that the prepared Ag-NPs can represent an alternative or auxiliary material used for controlling important nosocomial pathogens. The fungal reference strain *Candida albicans* was more sensitive at Ag-NPs actions (zone of inhibition = 20 mm) compared with the clinically isolated strain (zone of inhibition = 10 mm), which emphasizes the greater resistance of fungal strains at antimicrobial agent’s action.

## 1. Introduction

Silver nanoparticles (Ag-NPs) have attracted attention over time due to their physical, optical, electrical, and chemical properties and due to their multiple applications [1,2,3,4]. The improvement of mechanical, catalytic, and biological characteristics has been exploited in various fields, including catalysis, electronics, imaging, photonics to bio-sensitivity, and drugs. It is known that particles with dimensions smaller than 100 nm attract special attention due to the wide range of applications in industrial or medical fields [5].

Silver nanoparticles (Ag-NPs) represent one of the highly synthesized classes of nanoparticles with multiple applications [6]. In particular, thanks to the antimicrobial properties of Ag-NPs, they have been incorporated into many consumer goods, such as plastics, textiles, cosmetics, and food packaging materials, as well as in medical products such as dressings, implants, or catheters [7].

Through time, Ag-NPs have been synthesized using different methods: (i) chemical methods (reduction, electrochemical, hydrothermal, microemulsion, photochemical), (ii) physical methods (arc discharge, evaporation-condensation, high-pressure magnetron sputtering, laser ablation), and (iii) biological (algae, bacteria, fungi, plants, yeast) [8,9,10,11].

Each method has its advantages and disadvantages, but in almost all cases, the main problems are costs, particle size, and size distribution. Of the existing methods, the chemical reduction is the most widely used, as it provides an easier way to synthesize Ag-NPs. In general, the process of chemical reduction uses the following three components: (i) metal precursor (usually AgNO_3_) and (ii) reduction agents such as ethylene glycol, citrate, ascorbic acid, glucose, sodium borohydride, polyvinylpyrrolidone (PVP), and (iii) stabilizing/coating agents (e.g., citrate, polyvinylpyrrolidone (PVP), polyvinyl alcohol (PVA), sodium oleate [3,5,12,13,14,15,16,17,18,19,20]). A very powerful tool that can be used for the synthesis of inorganic materials with bio-properties has been biomimetically synthesized [21,22].

The formation of colloidal solutions by the chemical reduction of silver salts involves two stages: (1) the formation of nuclei and (2) the subsequent growth. It has also been found that the size and shape of the synthesized nanoparticles are highly dependent on these steps. In addition, for the synthesis of monodispersed Ag-NPs, with a uniform size distribution, it is necessary for all nuclei to form at the same time. In this case, all nuclei will have the same or similar size and will also have similar subsequent growth.

The initial nucleation and subsequent growth of the initial nuclei can be controlled by adjusting the reaction parameters such as temperature, pH, precursor: reducing agent: stabilizing agent ratio [3,14,15,17,19,20].

Many methods are aimed at the synthesis of Ag-NPs using organic molecules, such as polymer, to produce in-situ reducers. It is important that such macro-molecules interact with the silver ions, acting as anti-agglomeration agents, stabilizing the Ag-NPs [12]. The natural organic polymers used for this purpose are sucrose, maltose, chitosan, gum Arabic, plant extracts, and starch (an environmentally friendly and inexpensive biopolymer).

Usually, polymer-silver nanocomposites are produced, which are promising functional materials for different practical applications. Such nanocomposites are prepared by including silver nanoparticles into different polymer matrices using different routes [23]. For example, starch and Ag-NP solutions used in the paper industry for coating are prepared in several stages: the starch solution has been prepared by heating and stirring the mixture; after complete dissolution of starch, silver nitrate was added. The reduction of metallic ions was ensured using different energy sources such as heat, microwave, ultrasonication, or UV light [3,23].

In the present study, we studied the synthesis of Ag-NP particles starting from soluble starch and silver nitrate, with no use of any additional reducing agents, by ultra-sonication. Soluble starch is an eco-friendly and inexpensive reducing agent because hydrolysis processes lead to glucose molecule release. The novelty of the present paper is represented by the usage of starch as a reducing and stabilizing agent.

Starch hydroxyl groups are responsible for the complexation of silver ions, controlling the nanoparticle shape and size, leading to a good dispersion into the reaction mass [3,23,24,25,26,27].

Ultra-sonication is an effective method for colloidal silver nanoparticle preparation because it generates heat, improves starch dissolution, disperses the silver nitrate molecules, and is also an efficient reducing agent for silver ions. Ultra-sonication is a method used to obtain the proper dispersion of different materials involving physical-chemical interactions and different effects from clusters breaking and deagglomeration to chemical reactions [28].

Due to their intrinsic properties and multilayer actions, colloidal Ag-NPs have a broad spectrum of antimicrobial activity against different types of microorganisms, demonstrating a huge potential against even antibiotic-resistant microorganisms [29,30,31,32,33].

Ag-NPs are able to physically interact with the cell membrane of a broad spectrum of microorganisms (bacteria, fungi, etc.). Ag-NPs adhere and accumulate onto the surface of the microorganism’s cell membrane. Starting from the structure of the cell membrane and the different interactions of Ag-NPs with the cell membranes of the microorganisms, this study highlights the antimicrobial activity of the synthesized material compared with silver ions from an AgNO_3_ solution.

Our study is in concordance with the literature, showing that the new synthesized Ag-NPs don’t present a considerable inhibition zone, but such materials present a broad spectrum of bactericidal, fungicidal [34,35], and antiviral effects [36]; thus, making them ideal in biomedical usages, such as wound dressings, dentistry [37], and cancer treatments [38].

## 2. Materials and Methods

### 2.1. Ag-NPs Synthesis and Characterization

For the synthesis of the colloidal Ag-NPs were used: soluble starch (Merck, Germany), DI water, AgNO_3_ (Merck, Germany), and NaOH (Merck, Germany).

The characterization of the samples has been performed by: UV-VIS spectroscopy, using Varian Cary 50 spectrometer (Varian Inc., Santa Clara, CA, USA), scanning electron microscopy, SEM, energy-dispersive X-ray spectroscopy, EDX, using Quanta FEG 250 Scanning Electron Microscope (FEI, Hillsbro, OR, USA) and Fourier-transform infrared spectroscopy, FT-IR, using Bruker Platinum ATR-QL spectrometer (FTIR, Bruker, Billerica, MA, USA).

In order to gain information regarding the stability and electrical charge of silver nanoparticles, the zeta potential was determined using a Zeta-Meter System 3.0+, (Zeta_Meter, Inc., Staunton, VA, USA). Calibration of the apparatus was performed with a suspension standard of Min-U-SIL 100 mg/L (Merck, Darmstadt, Germany) in 100 mg/L NaCl solution. The measured zeta potential represents an average value of the results obtained after 10 readings for each sample. All samples were diluted in a ratio of 1:100.

### 2.2. Microbiological Activity

The antimicrobial activities of the Ag-NPs (synthesized at optimum pH) and Ag ions were determined in triplicate by the diffusimetric method according to the CLSI methodology (Clinical Laboratory and Standards Institute Inc., Wayne, PA, USA) [39].

The tested microorganisms included 5 reference strains from an American Type Culture Collection (ATCC): *Escherichia coli* (ATCC 25922) (Microbiologics, St. Cloud, MN, USA), *Pseudomonas aeruginosa* (ATCC 27853) (Microbiologics), *Staphylococcus aureus* (ATCC 25923) (Microbiologics), *Streptococcus pneumoniae* (ATCC 49619) (Microbiologics), *Candida albicans* (ATCC 90028) (Microbiologics), and 5 strains isolated from clinical cases (No. 25 / 06.12.2019, University of Oradea): *Staphylococcus aureus, Escherichia coli, Klebsiella pneumoniae, Enterococcus fecalis,* and *Candida albicans*.

Each strain was placed on its appropriate growth media: Mueller-Hinton agar (Oxoid, Hampshire, UK) for *Staphylococcus sp., Escherichia coli, Pseudomonas aeruginosa,* and *Enterococcus fecalis*. Mueller Hinton 2 (Oxoid, Hampshire, UK) supplied with 5% sheep blood (BioMerieux) agar media was used for the strain of *Streptococcus pneumoniae*, and Sabouraud Gentamicin Chloramphenicol 2 (BioMerieux, Marcy l’Etoile, France) agar media for the strains of *Candida albicans*. A standardized inoculum corresponding to an optical density of 0.5 McFarland units was used. Sterile filter paper discs with a diameter of 6 mm (HiMedia Laboratories, Mumbai, India) impregnated with 20 µL of positive control test solution (different antibiotics) were deposited onto the surface of the inoculated media. The plates were incubated at a temperature of 310 K for 24 h for the bacteria, respectively 310 K for 24 h plus 24 h more at 298 K for *Candida albicans*.

## 3. Results and Discussion

### 3.1. Ag-NPs Synthesis and Characterization

(a) Ag-NPs synthesis

For the synthesis of the colloidal Ag-NPs, 1 g of soluble starch was dissolved into 25 mL of DI water and stirred for 30 min at 328 K. Concomitant 0.250 g of AgNO_3_ was dissolved into 5 mL of DI water, and after complete dissolution of starch, AgNO_3_ solution was added and left to stir for approximately 10 min. Before subjecting the reaction mass to ultra-sonication, the pH was adjusted in the range of 8–13 by adding NaOH. Finally, the obtained samples were ultra-sonicated in the dark, at 353 K, for 60 min.

(b) UV-VIS spectroscopy analysis

UV-VIS characterization confirmed the reduction of silver ions to Ag-NPs.

**Figure 1:**
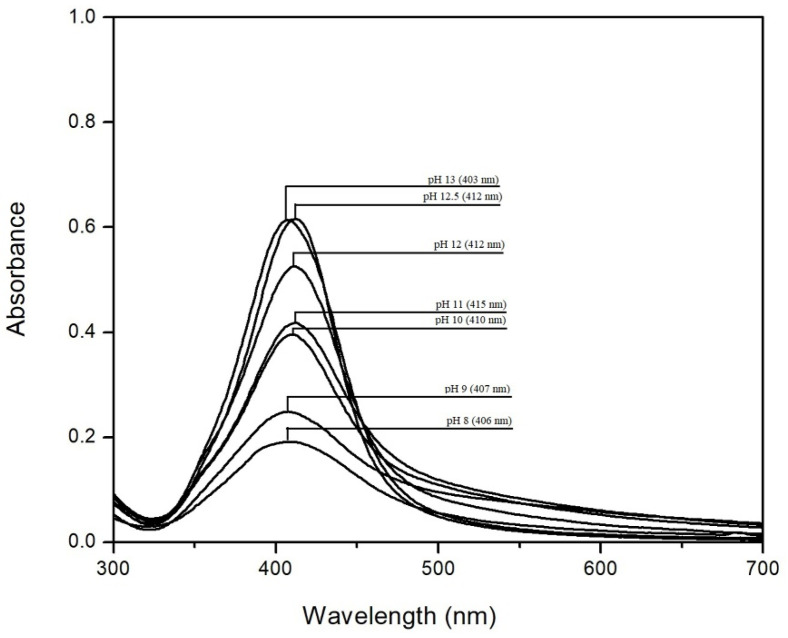
UV-VIS spectra recorded for prepared Ag-NPs.

Because pH has an important role in the synthesis of colloidal Ag-NPs, in order to track the influence of pH on Ag-NP synthesis, UV-VIS spectra were performed for the samples at different pH values, in the range of 8–13, which are shown in Figure 1.

The absorption band in the visible range from 350 nm to 550 nm highlights the presence of Ag-NPs formed. The peak appears at different wavelengths between 403–415 nm, depending on the pH [40,41,42].

It can be seen that with the increase of pH, the intensity of the absorbance increases, which also indicates an increase in the concentration of Ag-NPs [43]. At pH < 10, absorption bands are large and have a low height, which can be associated with an uneven particle distribution and a low concentration of Ag-NPs [44]. From the literature data, we found that the precipitation of silver ions with a formation of AgOH starts at a pH between 7 and 8 and stops at a pH around 13, while any further pH increase leads to precipitate dissolution [45]. At pH values greater than 10, the peaks from the UV-VIS spectra become much taller and sharper, which can be associated with a uniform distribution and higher concentration of Ag-NPs [12,44,46]. This observation is aligned with the data observed from SEM images. These data are in concordance with the information obtained from the EDX spectra, which confirm that the increase of pH leads to an increase in Ag-NP concentration.

From Figure 1, we observe that by increasing the pH to 12, it is possible to produce nanoparticles with higher stability, having smaller dimensions and uniform distribution. Furthermore, we show that at pH > 12, the absorption peak is sharper and more symmetrical, which can signify that the particles are smaller with uniform distribution. However, from the SEM images, it can be seen that at pH > 12, there is a tendency for particle agglomeration. Starting at pH 12.5, the absorbance remains approximately constant.

It is a fact that by solubilizing starch at higher temperatures, under stirring, starch hydrolysis with formation of glucose, and aldehyde groups oxidation with the formation of carboxylic groups, occurs. Furthermore, the increase of pH values leads to an increase of the hydroxyl groups with the formation of hydrogen bonds, facilitating the formation of Ag-NPs [1].

The generally accepted mechanism for Ag-NPs preparation is a two-step process. The first step is the reduction of Ag ions by glucose molecules formed during starch hydrolysis. In the second step, formed silver atoms act as nucleation centers, catalyzing the reduction reaction of remaining silver atoms. Subsequently, metallic clusters are formed by the association of silver atoms. Since, during the formation of such associations, the binding energy between different particles (metallic atoms or metallic ions) are involved, the superficial ions are again reduced, and the aggregation process continues until larger particles are obtained. Further, the nucleation process is stabilized by the interaction with the polymer, preventing particle coalescence and aggregation [1,47,48].

**Figure 2:**
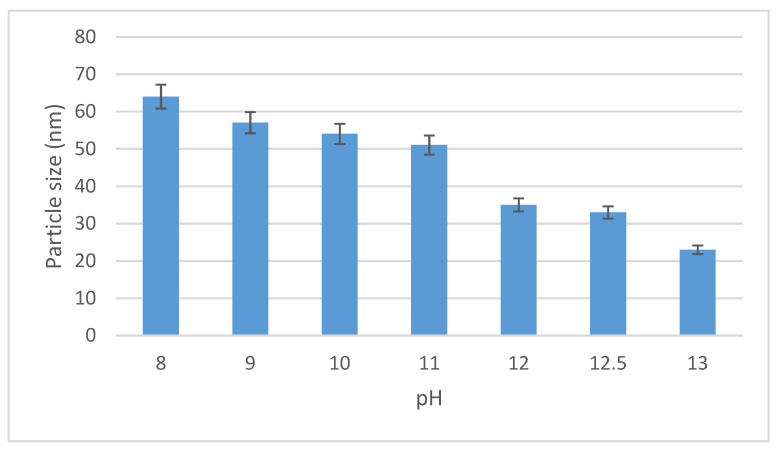
Particle size distribution of synthesized Ag-NPs vs. pH.

Simultaneously, with the increase of the pH, we noticed a color change from colorless to brown, along with the decrease of the particle size (data are depicted in Figure 2).

(c) Scanning electron microscopy, SEM, and studies

**Figure 3:**
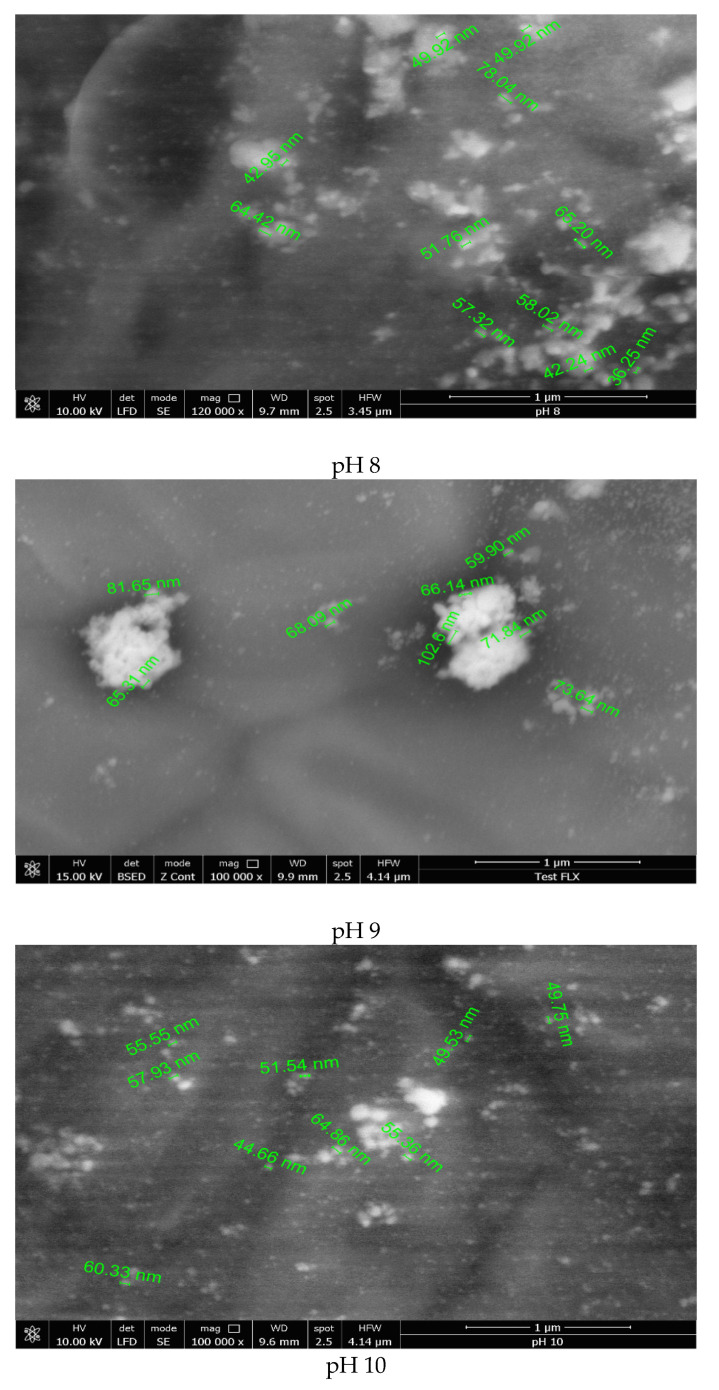

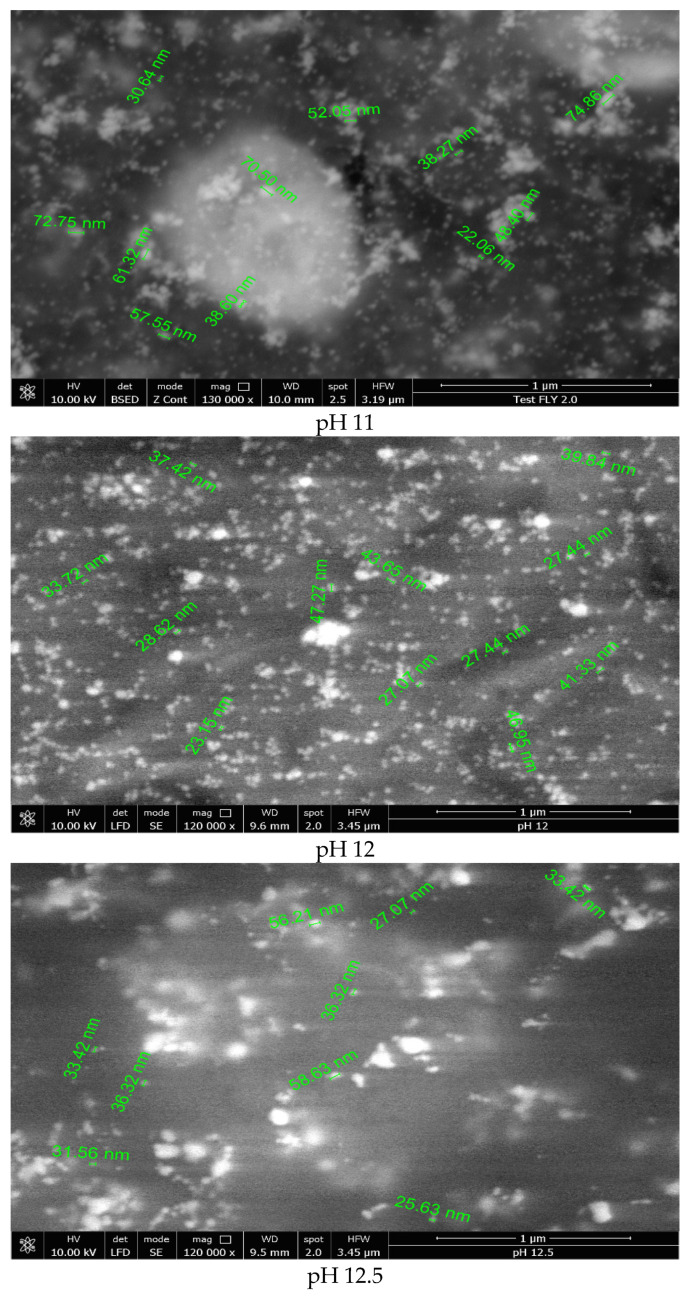

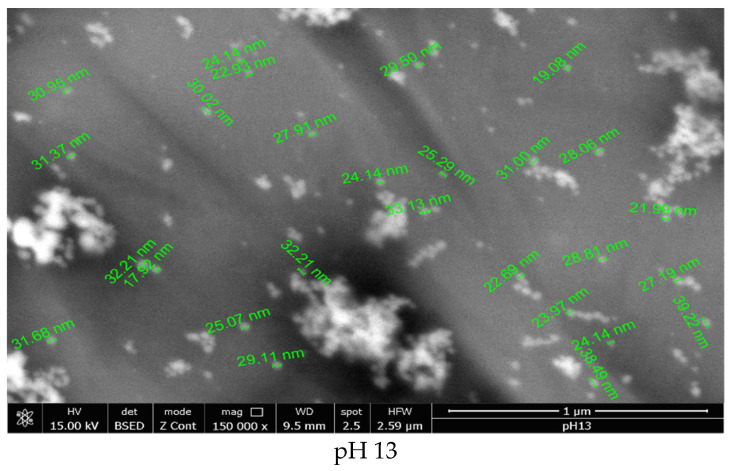
Scanning electron micrographs recorded for samples prepared at pH values between 8 and 13.

To investigate the large-scale morphology of Ag-NPs, the samples synthesized at different pH values were characterized by scanning electron microscopy, SEM (Figure 3).

According to the literature, the synthesized nanoparticles are spherical, symmetrical, and small [20,44]. From the data presented in Figure 3, the synthesized silver nanoparticles are present in an irregular shape when the reaction mass pH was lower than 11. This can be explained if we consider the lower rate of starch hydrolysis and nucleation, leading to the formation of nanoparticles with higher dimensions and irregular shapes [29]. Contrariwise, it was observed that at pH > 11, most of the nanoparticles present as regular spherical shapes, with dimensions in the range of 20–60 nm.

Based on the data obtained from UV-VIS spectra and SEM images, the optimum pH for the synthesis of Ag-NPs is 12. At pH > 13, the formation of [Ag(OH)_2_]^−^ species takes place, determining the modification of silver ion reduction potential as well as the destabilization of colloidal silver nanoparticles.

(d) Energy-dispersive X-ray spectroscopy analysis, EDX

**Figure 4:**
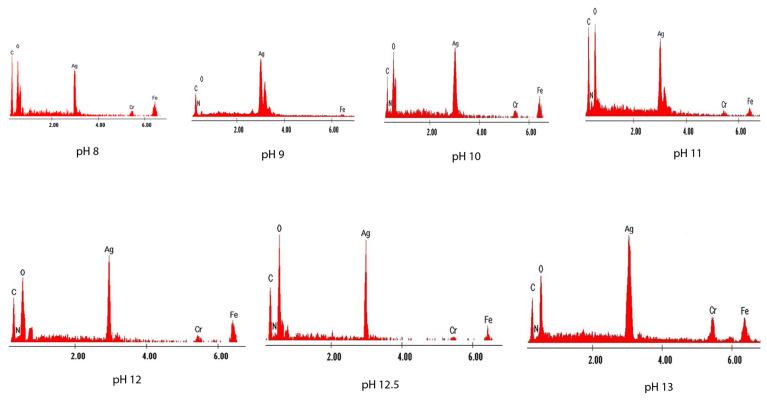
Energy-dispersive X-ray spectra recorded for samples prepared at pH values between 8 and 13.

In order to highlight the presence of silver in the synthesized materials, energy-dispersive X-ray spectroscopy spectra were obtained (Figure 4). The spectra show the emissions of characteristic X-rays from analyzed samples. The height of each specific peak is proportional to the quantity of the specific atom. To achieve such characteristic X-ray emissions, component atoms of samples were excited using an electron fascicle.

Regardless of the pH value, the silver peak can be observed in the EDX spectra, noting that the amount of silver increases with the pH, which also confirms the morphology shown in the SEM images [3,44,49,50,51,52].

(e) Fourier transform infrared spectroscopy analysis, FT-IR

**Figure 5:**
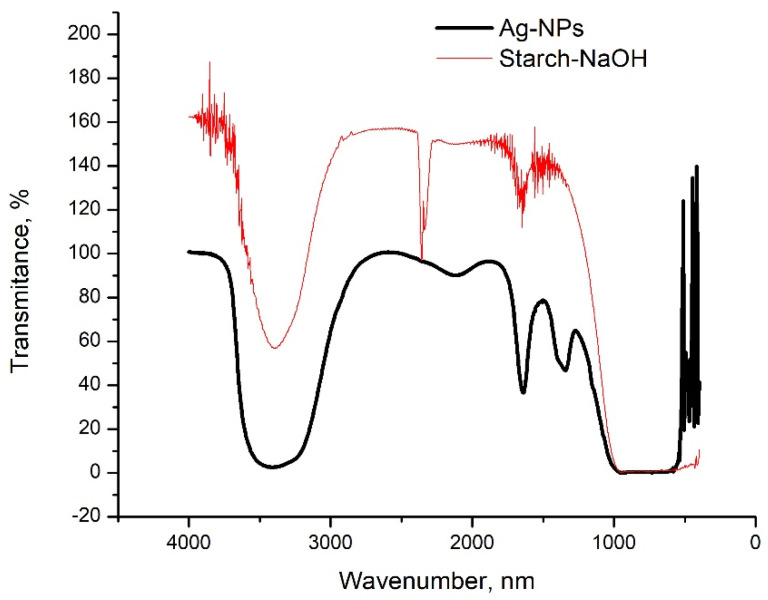
FT-IR spectra were recorded for Ag-NPs and Starch-NaOH.

The FT-IR spectra for the colloidal Ag-NPs and the starch solution, which only had the pH adjusted, without the addition of AgNO_3_, are shown in Figure 5.

In both spectra, in the range of 3200–3550 cm^−1^, a vibration appears specific to the O-H bond. Moreover, at the wavenumber 2121 cm^−1^, a peak specific to the vibration of the aliphatic C-H bond appears. More significantly, a peak specific to the vibration of the C=O bond appears in the sample with starch and NaOH only, at the wavenumber of 1645 cm^−1^.

At the wavenumber 1352 cm^−1^, a peak specific to the vibration of the carboxyl group COOH appears, which is highlighted only in the Ag-NPs spectrum. This vibration is due to the binding of the Ag-NPs to the surface through the carboxyl group, and according to the literature data, this is specific to the synthesis of Ag-NPs [12,16,17,53].

(f) Zeta potential of Ag-NPs measurement

For characterization of the colloidal particles, there is no satisfactory technique to evaluate their superficial charge. The usual practice is to determine the electrical potential of a particle from the diffuse layer, away from the particle surface. This plane, in which the particle moves, is called the slip plane or the shear plane. The measurement of the potential for this plane is called the zeta potential, which is an important parameter for colloidal nanoparticles. The zeta potential value is closely related to the suspension stability and to the particle surface morphology, but also to the interaction potential of the nanoparticles with the microbial cell wall membrane [54].

The zeta potentials of Ag-NPs, depending on the pH value, are presented in Table 1.

From the obtained data from the zeta potential, results show that the nanoparticle charge is directly influenced by the pH value. Thus, in the range of 8–10, the zeta potential has positive values; therefore, the nanoparticles are positively charged. At the same time, the medium nanoparticle size is between 40 and 80 nm.

In the range of 11–13, the zeta potential has negative values, thus indicating that the nanoparticle charge is also negative, and the medium sizes of these nanoparticles are between 20 and 75 nm.

Solutions with a zeta potential less than −30 mV or greater than +30 mV have a higher level of colloidal stability [55]. At pH > 12, the zeta potential has a value of +30 mV, and silver nanoparticles are uniformly dispersed, as can be seen from the SEM. At pH > 13, silver nanoparticles tend to agglomerate due to the apparition of [Ag(OH)_2_]^−^ species into the system [45]. However, it is important that the oxidation process of carbonyl groups take place at a higher rate in order to cover the electrons needed to reduce silver ions. Increasing the pH leads to an increase in starch solubility, providing fragments with higher affinity and reduction capacity required during the synthesis of stable Ag-NPs colloids. Soluble starch macromolecules are composed of branched amylopectin groups. By increasing the pH, these macromolecules break into glucose fragments, which have the ability to reduce the silver ions [1].

Standard reduction potential for silver reduction is +800 mV (corresponding to the reaction Ag^+^ + e^−^ → Ag(s)). At pH 7 to 8, the hydrolysis of silver ions takes place:

2Ag^+^(aq) + 2HO^−^ → 2AgOH(s) → Ag_2_O(s) + H_2_O

In this case, the reduction potential has a value of +344 mV, corresponding to the following reaction [1]:

Ag_2_O(s) + H_2_O + e^−^ → 2Ag_(s)_ + 2HO^−^

Glucose oxidation is taking place at a potential 750 mV. In this case, the global reaction associated with silver nanoparticle synthesis is:

C_6_H_12_O_6_ + 2AgOH_(s)_ → C_6_H_12_O_7_ + 2Ag_(s)_ + H_2_O

### 3.2. Microbiological Activity

Because a growing number of bacteria, fungi, and viruses are resistant to all or almost all currently available drugs, it is necessary to develop new antimicrobial agents with superior antimicrobial properties, to target a wide range of microorganisms [56]. A possible way to develop new antibacterial agents is represented by the production of some viruses (such as phage or phage-modified viruses) that are able to specifically infect bacteria and are used in antibacterial therapy [57,58]. The usage of Ag-NPs represents an alternative method to treat bacterial infection due to the lack of side effects.

This is the reason why, in this paper, we show that the newly synthesized material, based on silver nanoparticles, has increased antimicrobial potential compared to Ag ions in the AgNO_3_ solution. Thus, this new material can have application in the microbiological field. For these, we tested the effectiveness of the newly synthesized material, inoculated in bacterial and fungal cultures, to demonstrate that this newly obtained material has antimicrobial activity, both antibacterial and antifungal.

To assess the antimicrobial activity, the diameter of the inhibition zone was measured in mm using a ruler. Each test was performed in triplicate, and the results were expressed as an arithmetic mean.

Standard Ciprofloxacin (5 µg; Oxoid), Cefoxitin (30 µg; Oxoid), Erythromycin (15 µg; Oxoid) and Caspifungin (5 µg; Oxoid) discs were used as a positive control, and discs impregnated with 20 mL of distilled water as a negative control.

The antimicrobial activity was tested upon five reference strains belonging to the American Type Culture Collection, *Staphylococcus aureus* (ATCC 25923), *Pseudomonas aeruginosa* (ATCC 27853), *Escherichia coli* (ATCC 25922), *Pseudomonas aeruginosa* (ATCC 27853), *Streptococcus pneumoniae* (ATCC 49619), and *Candida albicans* (ATCC 90028) along with five clinically isolated strains *Staphylococcus aureus, Meticilina Sensibile*, *Enterococcus fecalis, Escherichia coli, Klebsiella pneumoniae,* and *Candida albicans*.

In the case of reference strains used as a positive control in the testing of the synthesized material, the values obtained were compared with the interval recommended by the Clinical and Laboratory Standards Institute (CLSI) for disk diffusion tests [39].

The interval framing shows that the method was correct, and these strains are quality control strains. In the case of clinically isolated strains, we reported the obtained values, and in parallel, we specified the value from which, according to CLSI, the strains are considered sensitive [39].

Table 2 shows the obtained inhibition diameters measured on Petri dishes following microbiological tests on reference strains compared with the control test and standards from CLSI.

Table 3 shows the obtained inhibition diameters, measured on Petri dishes following microbiological tests on clinically isolated or antibiotic-resistant strains, compared with the control test and standards from CLSI.

Two solutions were used: AgNO_3_ solution, which represents the precursor of the synthesis, and the colloidal solution of Ag-NPs.

As can be seen, regardless of all the tested microorganism types, the inhibition diameter corresponding to the Ag-NPs solution is larger than those related to the AgNO_3_ solution. It is therefore obvious that the size of the Ag-NPs has a particularly important role in the exercise of the antimicrobial function, which, in the case of the sample with pH = 12, are about 20–50 nm in size. The important role of the Ag-NPs size and the specific surface area has been highlighted in various studies [59,60].

In the case of the tests for *Escherichia coli* and *Staphylococcus aureus*, while analyzing the inhibition diameters resulted from the tests done with Ag-NPs, in both the microbiological cultures (the reference bacterial and clinically isolated strains), it was observed that they didn’t differ substantially. Therefore, we can conclude that the synthesized material has significant biocidal properties. In the case of *Staphylococcus aureus,* we can observe that the clinically isolated strain has a similar bactericidal response compared to the reference strains because the inhibition zone diameter is 19 mm in both cases.

In the case of *Escherichia coli,* the clinically isolated strain has a better bactericidal response (18 mm) compared to the reference strains (16 mm).

As clinically isolated bacterial strains are normally resistant to current antibiotics, the inhibitory disc would be expected to be smaller in diameter if the clinically isolated bacteria were also resistant to the synthesized material [61].

Based on this argument, we claim that the clinically isolated strains used in our tests have a lower resistance to newly synthesized material, and therefore have potential use in the medical field.

We can also see that the newly synthesized material showed an antibacterial activity on both Gram-positive and Gram-negative bacteria, even if it is slightly lower than the bactericidal activity observed in the antibiotics used as controls. One explanation would be the interaction of silver ions with the surface of the bacterial cell wall and the enzymatic thiol groups, which seem to be the first step in the degradation of the bacterial cells [59].

In this context, we consider that the tested material can be used on a complex bacterial consortium consisting of several types of bacteria, with very good bactericidal results. However, the results suggest that the synthesis of the new Ag-NP material could be considered an innovative, simple, and viable strategy to obtain a promising antibacterial solution. This is assuming the synthesized material is non-toxic to the environment and has no adverse effects as antibiotics do after long usage.

Regarding the antifungal effect of the synthesized material, in the case of Ag-NP material used on the reference culture *Candida albicans* (ATCC 90028), the inhibition diameter of 20 mm is comparable to that presented in the specialized protocol CLSI 2020 for the antibiotic CASPOFUNGIN 5 µg (22 mm). This falls within the range of sensitivities of the standardized antibiotic, which proves our material to be effective regarding its antifungal properties.

In the tests on clinically isolated *Candida albicans* strains, the results are not as promising due to the fact that the strain has a special resistance to the action of antifungal agents due to its dimorphic structure, rapid adaptation to pH fluctuations, metabolic flexibility, and the presence of a strong nutrient-acquiring system from the environment [62].

In conclusion, fungal infections remain a very serious and common disease, for which active or more concentrated antifungal agents are needed in order to control them properly. We can not exclude that by improving the properties of this material (particle size, shape, pH, or solution concentration), the antifungal activity would be improved.

## 4. Conclusions

In conclusion, this study demonstrated a simple, clean, economical, non-toxic, and environmentally-friendly technique by which colloidal Ag-NPs can be synthesized. This method uses indirect ultra-sonication with soluble starch as a stabilizer and reducer for silver nanoparticle synthesis. The reducing agent was obtained from the hydrolysis of amylopectin chains from starch macromolecules.

We also showed the relationship between the pH values and nanoparticles size. Thereby, the chosen method demonstrated the preparation of spherical silver nanoparticles with dimensions between 20 and 80 nm.

The colloidal Ag-NPs were obtained using physico-chemical characterizations, including UV-VIS spectroscopy, scanning electron microscopy, energy-dispersive X-ray spectroscopy analysis, and Fourier transform infrared spectrometry.

An increase in the solution pH caused important modifications in the UV-VIS spectra. Therefore, the pH is directly related to the silver nanoparticle stability. Superficial plasmonic excitations observed at the Ag-NPs surface appear at variable wavelengths between 402 and 415 nm. The highest adsorption peak was observed for the silver nanoparticles obtained at pH values between 12 and 12.5. In this case, the silver nanoparticles present the lowest size and uniform distribution.

The pH influence on the electrical charge of the particle surface (related to the stability of the colloid) was highlighted by the determination of zeta potential, observing that the increase of pH leads to zeta potential increase. For the particles obtained at pH 12, the zeta potential is higher than 30 mV, which is related to the higher stability of the colloidal system. At pH > 12 and high zeta potentials, agglomeration tendency has been observed due to the formation of [Ag(OH)_2_]^−^ ions.

From a structural point of view, as the nanoparticle size decreases and the surface-volume ratio increases, which results in better adhesion of Ag-NPs to the surface of the bacterial cell wall, thus increasing the antimicrobial effect of the synthesized Ag-NPs. The increased antimicrobial effect in the case of the material with Ag-NPs compared to the AgNO_3_ solution is proved by comparing the size of the inhibition zone diameters between the two samples for the same microbial strain. In all tests performed, the antimicrobial response, expressed by the corresponding inhibition diameter, is superior to the Ag-NP solution. Thus, samples with Ag-NPs show antimicrobial activity that is good (for *Staphylococcus aureus*) or moderate (the other tested strains), but it is always superior to that manifested by Ag^+^ ions from the AgNO_3_ solution.

The clinically isolated strains of bacteria used in our tests have a lower resistance to newly synthesized material, which is a desideratum to consider our material as having potential use in the medical field.

The results obtained from the clinically isolated strain are not as promising due to the fungus’ resistance to the antifungal agents, based on its dimorphic structure, rapid adaptation to environmental pH fluctuations, and metabolic flexibility. However, the references strain of *Candida albicans* has a very good sensibility with our synthesized material.

The study of Ag-NP synthesis mechanisms remains a challenge because small variations in the synthesis conditions lead to modifications of size, shape, and composition of prepared nanoparticles.

## Figures and Tables

**Table 1:** The zeta potential of Ag-NPs, based on the pH value.

pH	8	9	10	11	12	12.5	13
Zeta Potential, mV	15.8	11.6	7.14	−15.1	−37.9	−51.4	−70.5

**Table 2:** The inhibition diameter of AgNO_3_ solution and Ag-NPs solution on different strains of microorganisms.

Indicator Strains	Zone of Inhibition (mm)				Antibiotics Used in Positive Control Tests
	AgNO_3_ Solution	Ag-NPs	Positive Control Test	Standard CLSI	
*Staphylococcus aureus* ATCC 25923	11	19	26	23–29	CEFOXITIN 30 µg
*Pseudomonas aeruginosa* ATCC 27853	9	16	27	25–33	CIPROFLOXACIN 5 µg
*Escherichia coli* ATCC 25922	9	16	37	29–38	CIPROFLOXACIN 5 µg
*Streptococcus pneumoniae* ATCC 49619	8	11	30	25–30	ERITROMICINA 15 µg
*Candida albicans* ATCC 90028	10	20	22	18–27	CASPOFUNGIN 5 µg

**Table 3:** The inhibition diameter of AgNO_3_ solution and Ag-NPs solution on different clinically isolated strains.

Clinically Isolated Strains	Zone of Inhibition (mm)				Antibiotics Used in Positive Control Test
	AgNO_3_ Solution	Ag-NPs	Positive Control Test	Standard CLSI	
*Staphylococcus aureus Meticilina Sensibil* (MSSA)	10	19	24	S ≥ 22	CEFOXITIN 30 µg
*Enterococcus faecalis*	10	18	23	S ≥ 21	CIPROFLOXACIN 5 µg
*Escherichia coli*	9	18	34	S ≥ 31	CIPROFLOXACIN 5 µg
*Klebsiella pneumoniae Ssp pneumoniae*	9	19	35	S ≥ 31	CIPROFLOXACIN 5 µg
*Candida albicans*	8	10	18	S ≥ 17	CASPOFUNGIN 5 µg

## Data Availability

Not applicable.
